# Why do some countries do better or worse in life expectancy relative to income? An analysis of Brazil, Ethiopia, and the United States of America

**DOI:** 10.1186/s12939-020-01315-z

**Published:** 2020-11-10

**Authors:** Toby Freeman, Hailay Abrha Gesesew, Clare Bambra, Elsa Regina Justo Giugliani, Jennie Popay, David Sanders, James Macinko, Connie Musolino, Fran Baum

**Affiliations:** 1 Southgate Institute for Health, Society, and Equity, Flinders University, Adelaide, Australia; 2 Department of Public Health, Flinders University, Adelaide, Australia; 3 Department of Epidemiology, Mekelle University, Mekelle, Ethiopia; 4 Institute of Population Health Sciences, Newcastle University, Newcastle, UK; 5 Pediatrics Department, Universidade Federal do Rio Grande do Sul, Porto Alegre, Brazil; 6 Division of Health Research, Lancaster University, Lancashire, UK; 7 School of Public Health, University of the Western Cape, Cape Town, South Africa; 8 Departments of Health Policy and Management and Community Health Sciences, UCLA, Los Angeles, CA USA

**Keywords:** Population health, Social determinants of health, Policy, Life expectancy, Civil society

## Abstract

**Background:**

While in general a country’s life expectancy increases with national income, some countries “punch above their weight”, while some “punch below their weight” – achieving higher or lower life expectancy than would be predicted by their per capita income. Discovering which conditions or policies contribute to this outcome is critical to improving population health globally.

**Methods:**

We conducted a mixed-method study which included: analysis of life expectancy relative to income for all countries; an expert opinion study; and scoping reviews of literature and data to examine factors that may impact on life expectancy relative to income in three countries: Ethiopia, Brazil, and the United States. Punching above or below weight status was calculated using life expectancy at birth and gross domestic product per capita for 2014–2018. The scoping reviews covered the political context and history, social determinants of health, civil society, and political participation in each country.

**Results:**

Possible drivers identified for Ethiopia’s extra 3 years life expectancy included community-based health strategies, improving access to safe water, female education and gender empowerment, and the rise of civil society organisations. Brazil punched above its weight by 2 years. Possible drivers identified included socio-political and economic improvements, reduced inequality, female education, health care coverage, civil society, and political participation. The United States’ neoliberal economics and limited social security, market-based healthcare, limited public health regulation, weak social safety net, significant increases in income inequality and lower levels of political participation may have contributed to the country punching 2.9 years below weight.

**Conclusions:**

The review highlighted potential structural determinants driving differential performance in population health outcomes cross-nationally. These included greater equity, a more inclusive welfare system, high political participation, strong civil society and access to employment, housing, safe water, a clean environment, and education. We recommend research comparing more countries, and also to examine the processes driving within-country inequities.

**Supplementary Information:**

The online version contains supplementary material available at 10.1186/s12939-020-01315-z.

## Background

Life expectancy ranges from 52 years in Sierra Leone and the Central African Republic to 84 years in Japan and Hong Kong [1] – a staggering gap of 32 years. These extreme health inequities partly reflect wealth inequities between countries. Generally, wealthier countries have a higher average life expectancy than poorer countries [2–4], which can be argued to be achieved through higher standards of living, more effective health systems, and more resources invested in determinants of health (e.g. sanitation, housing, education) [5]. Preston [3] found that there was a cross-sectional logarithmic curve relationship between national income per capita and life expectancy, such that life expectancy rapidly increases with national income at first, until it begins to taper off, with higher income countries receiving diminishing returns for increases in national income. This “Preston curve” was a fitted trendline, with some countries falling above the trendline, achieving higher life expectancy than what would be expected from their income, and some countries falling below the trendline, achieving lower life expectancy than what their income would predict [3]. Thus, while it remains important to act on the drastic wealth inequities between countries, it is also important to consider why some countries have better or worse life expectancy than would be expected relative to their income. We use the term “punching above their weight” (PAW) [6] to describe those countries that achieve better population health outcomes and subsequently better life expectancy than would be anticipated from their wealth. Conversely, we use the term “punching below their weight” (PBW) for those countries that fail to translate their wealth into better population health and high life expectancy. Preston speculated on the drivers of countries punching above or below weight, hypothesising that within-country inequities would contribute to punching below weight [3].

Answers to the question of why some countries punch above or below weight are still partial, and this is the gap the research reported here sought to address. Previous research has largely focused on health outcomes in low and middle income countries (LMIC), and on the impact of the health system on health outcomes, giving less attention to other potential contributing factors [7, 8]. The seminal 1985 study ‘Good Health at Low Cost’ assembled a collection of expert reflections presented at a conference on the achievements of four low income countries who had achieved good health outcomes: Sri Lanka, China, Costa Rica and Kerala State in India [7]. The concluding conference statement identified five factors that supported these countries to achieve good health outcomes at a low cost: i) political commitment to advancing health, ii) valuing equity and community participation, iii) provision of quality education, especially for women, iv) sufficient and sustained investment in primary health care, and v) strong intersectoral linkages to support health [7].

A further study in 2011 followed up on the four countries from the Good Health at Low Cost study, and selected five new case study countries: Bangladesh, Ethiopia, Kyrgyzstan, Thailand, and the state of Tamil Nadu in India [8]. The researchers collected health indicator data, reviewed literature, and drew on theoretical frameworks to analyse the case studies. Their findings suggested that the development of strong and resilient health care systems supported by effective governance and agility during times of political unrest, conflict and natural disasters contributed to achieving substantial advances in health status [8]. While these studies noted the importance of transport infrastructure, gender equity and education, their focus was primarily on the role of health services. Broader social, environmental, political and commercial determinants of health and their interactions have received little attention. Studying these drivers in both punching above weight, and punching below weight countries may yield further insights into how countries can maximise their population health outcomes given their level of income.

Research has highlighted how population health is affected by a complex range of social, political, and commercial determinants of health [9, 10], which interact through a web of causations operating within and across interconnected systems [10]. Population health is affected by policies and actions in and beyond health care systems that act on the social determinants of health such as alleviating poverty and hunger; providing education, access to safe water, housing, sanitation and labour rights; and action on climate change [11]. This complex multifaceted set of long-term processes presents a challenge to research seeking to understand how to improve national population health outcomes. While Preston curves are typically examined as a cross-section of one point in time, countries’ historical legacies will differ, including factors such as colonisation, and the history of political institutions and their relationship to economic growth [12]. A country’s history is therefore critical to understanding contemporary economic and health outcomes.

The authors of this paper are members of *The Punching Above Weight (PAW) Research Network* (https://www.flinders.edu.au/southgate-institute-health-society-equity/punching-above-weight-network). The network was set up in 2017 to examine the central question of: why do some countries have better or worse life expectancy than would be expected relative to their gross domestic product (GDP)? [6]. The research reported in this paper aimed to assess the extent to which existing literature and data can answer the question through scoping reviews of three countries: Ethiopia (PAW), Brazil (PAW), and the United States (PBW).

## Methods

This paper used a mixed-methods approach involving: 1) quantitative analysis of life expectancy relative to national per capita income, 2) an expert opinion study, and 3) scoping reviews of existing literature and data to examine factors that may affect the relationship between life expectancy and GDP in our three case study countries: Ethiopia, Brazil and the United States. We then undertook a narrative synthesis of these three sources - quantitative indicators, expert opinion, and existing literature and data on the context, history, and policies- for each country [13] to identify possible driving factors of population health performance relative to GDP.

### Quantitative analysis

Punching above or below weight status was calculated in Microsoft Excel for each country from the Preston Curve (non-linear regression) for life expectancy at birth, and GDP per capita (International dollars, purchasing power parity) [3] using World Bank 2018 data [1, 14]. A country was deemed as PAW if they sat above the Preston Curve (i.e. had a higher life expectancy than would be predicted by their GDP, positive residual), and PBW if they sat below the regression curve (i.e. had a lower life expectancy than would be predicted by their GDP, negative residual). Since research has shown punching above or below weight status can change over time [6], we checked for persisting performance over time by calculating the Preston Curve for equivalent 2014, 2015, 2016, and 2017 data as well as for 2018.

### Expert opinion

To ensure our analysis focused on appropriate possible drivers of countries punching above or below weight, across low, middle, and high-income countries, we used an expert opinion study. Diverse global public health expert opinions were gathered through two formal meetings of the PAW Research Network. This Network comprised of academic experts, policy makers, and civil society actors. They had experience with research, civil society or policy on determinants of health and health inequities. A globally diverse membership was sought, including members from low, middle, and high-income countries. Experts were invited through the networks of the two key conveners who were both members of the People’s Health Movement, a global civil society group that focused on health equity. This aided them in achieving global coverage, and including low, middle, and high income country participants.

**Fig. 1:**
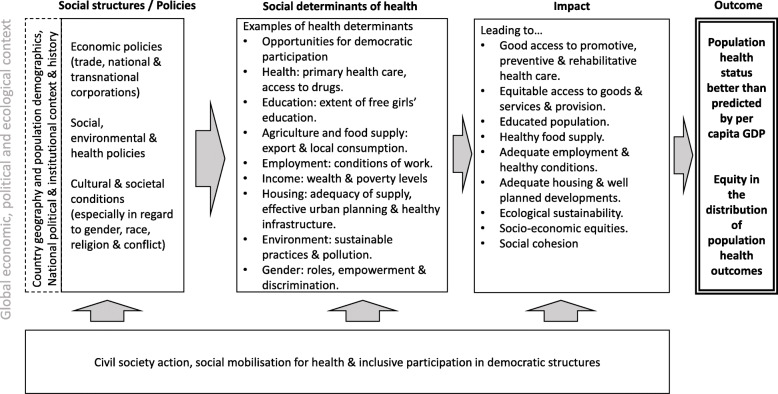
Framework for investigating why some countries punch above or below their economic weight in terms of life expectancy (adapted from Baum et al. [6])

The first meeting was held in Bellagio, Italy in 2017, and was attended by 21 members from 13 countries. The second meeting was held in Cape Town, South Africa in 2019 and was attended by 16 members from 8 countries. We felt the complexity and interactions between social determinants of health and life expectancy relative to national income precluded more structured methods such as Delphi and Nominal Group Technique, which aim to provide consensus rankings [15]. Instead, research presentations were provided by selected members on methodological considerations, conceptualising the research questions, and potential drivers of population health outcomes in selected countries. At the first meeting, this was followed by group brainstorming sessions to develop a theoretical framework to guide the research [6]. This framework was further refined during brainstorming sessions at the second meeting. The revisions included highlighting the importance of the global context, and the inclusion of equity in the distribution of population health outcomes within a country. This conceptual framework was the primary output of the expert opinion study (see Fig. 1). It includes the social structure and policies (e.g. social and economic policies), social determinants of health (e.g. extent of free girls’ education), their impact (e.g. educated population) and outcome for a given country (e.g. equity in distribution of health outcomes). The research reported in this paper represents the first application of the framework to country level data collection and analyses.

### Scoping review

#### Protocol

Scoping reviews were conducted of the available literature and data for Ethiopia, Brazil and the United States. Three was the minimum number that would allow inclusion of both PAW and PBW countries, and a low, middle, and high income country, while remaining a low enough number to allow in depth investigation of each country.

In choosing these three countries, we considered the following criteria: (i) availability of at least two members of the research network who have extensive knowledge of the literature, policies, and political and historical context in that country, to be lead and second researcher; (ii) a geographic spread and spread of country income categories; and (iii) inclusion of punching above and below weight countries. This resulted in the selection of Ethiopia (low income, PAW), Brazil (middle income, PAW) and the United States (high income, PBW). The reviews sought information on: 1) the political and institutional context and history of each country, 2) income, wealth and poverty, 3) education and gender, 4) healthy infrastructure (e.g. sanitation, public health regulation), 5) the health system, and 6) civil society organisation and political participation. We focused our search, analysis, and discussion on positive factors for the two PAW countries, and negative factors for the PBW country to answer our research question of what may be driving their punching above or below weight status. Because the spread of potential drivers of population health outcomes in each country was so broad, a systematic review of the literature on each driver in each country was not feasible. Instead, we used a scoping realist review approach [13] that sought to identify enough literature that contained information on plausible drivers across the range of domains contained in the study framework to feel confident that we were illuminating the key historic, contextual, and policy drivers that may have affected countries’ punching above or below weight outcomes. Preliminary results of the scoping reviews for each country were presented at the 2019 expert opinion meeting by lead researchers (Ethiopia: HG, Brazil: EG, United States: CB). Following expert opinion discussion, the reviews for each country were refined. All authors then reviewed revised analyses for each country.

#### Eligibility criteria

The following types of papers from the three countries were included: (i) qualitative and quantitative studies, (ii) short reports and (iii) guidelines, which assessed or described the aforementioned information.

#### Search strategy

We performed a comprehensive literature search of electronic databases Google Scholar, PubMed, MEDLINE, CINAHL and Web of Sciences, from 1997 (to constrain the search to recent factors that may explain current PAW or PBW performance) to December 2019. In addition, we searched several grey literature sources including the World Bank, World Health Organization, UNAIDS, and each country’s Ministry of Health reports and websites. Reference lists of obtained sources were also checked for relevant literature.

Search strings incorporated the following three concepts: health indicators and social determinants of health, life expectancy, and the country’s name. Table 1 lists the search terms for each concept. Searches used one country name and required at least one life expectancy term and at least one health indicator or social determinants of health term.

**Table 1:** concepts and search terms

Concept	Search terms
Health indicators and social determinants of health	political, economic, social, education, culture, gender, race, religion, health care, health system, primary health care, PHC, universal health coverage, UHC, health inequity, access to drugs, health indicators, infant mortality rate, IMR, under five mortality rate, child mortality, U5MR, CMR, maternal mortality ratio, maternal mortality rate, MMR, HIV, agriculture, food supply, import, export, employment, child labour, maternity leave, pension, purchasing power, social security, health care finance, insurance, community based health program, housing, access to safe water, sanitation, environment, pollution, sustainable practices, sustainability, urban planning or healthy infrastructure; gross domestic product or GDP
Life expectancy	life expectancy or longevity
Country	Ethiopia, Brazil or the United States (of America (US)).

#### Study selection and data extraction processes

We initially screened the title and abstract of each search result to ascertain if the reference provided information relevant to our research question – i.e. on potential drivers of good or poor population health outcomes. Then if relevant, we reviewed the full text to extract the relevant information. Authors HG, EG and CB led the literature search for Ethiopia, Brazil and the United States, respectively. We did not apply a formal critical appraisal to check the methodological quality or risk of bias for each study, but the lead authors used their own judgement of the quality of the article.

#### Synthesis

We synthesised relevant quantitative and qualitative information from the included articles. The framework guided us to thematize the relevant information. HG, EG and CB initially developed the themes and these were discussed by all team members which the following themes and subthemes were confirmed: 1) political and institutional context and history, 2) social determinants of health including education and gender; health system and infrastructure; income, wealth and poverty; housing; and environment, and 3) civil society organisation and political participation.

## Results

**Fig. 2:**
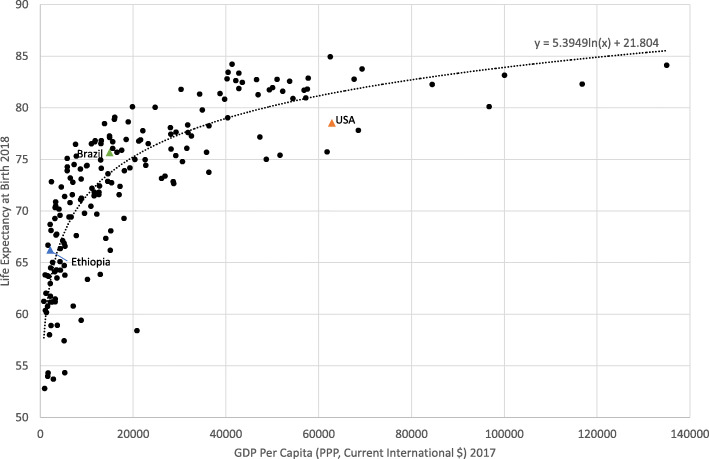
The relationship between life expectancy and gross domestic product per capita, 2017

Figure 2 shows the Preston Curve with 2018 data, and highlights the position of the three studied countries.

**Fig. 3:**
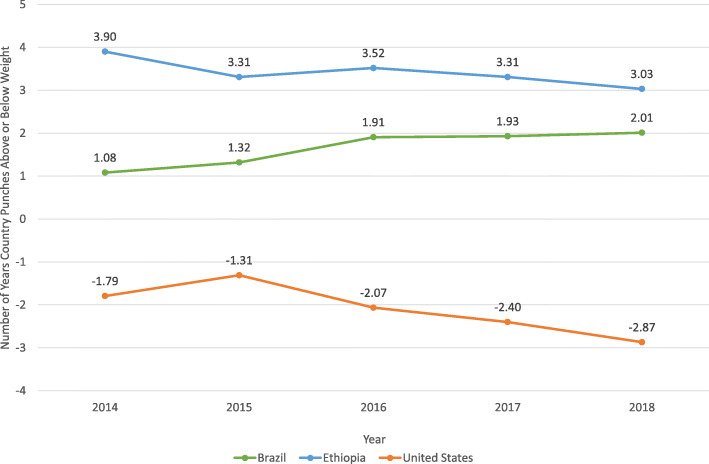
The number of years of life expectancy Ethiopia, Brazil, and the United States punched above or below weight for each year from 2014 to 2018]

Figure 3 shows the number of years Ethiopia, Brazil, and the United States punched above or below weight from 2014 to 2018 and their performance each year. Ethiopia and Brazil punched above weight in all years, while the United States punched below weight for all years.

Table 2 shows the life expectancy and selected key indicators of context and social determinants of health for each country. The sources for the table are available in a supplementary document (S1 doc). The findings are considered in relation to the countries’ income level (rather than the three countries being compared to each other).

**Table 2:** Punching above or below weight status and selected context and social determinants of health indicators of Ethiopia, Brazil and the United States of America, 1990s-2018

Characteristics	Ethiopia	Brazil	United States of America
Life expectancy at birth, 1997	50.3	68.6	76.4
Actual life expectancy at birth, 2018	66.2	75.7	78.5
Expected life expectancy at birth,^a^ 2018	63.2	73.7	81.4
GDP per Capita PPP, 2018	$2153	$14,941	$62,840
Life expectancy relative to GDP (PAW or PBW) status^b^	PAW, + 3.0 years	PAW, + 2.0 years	PBW, − 2.9 years
Context			
Country geography and population demographics	East Africa, 1.2mil km^2^, 100mil people (~ 80% are rural, 82% dependency ratio,^c^ male: female (M: F) ratio is 100:101), land locked	Latin America, 8.5mil km^2^,~ 210 mil people (~ 80% urban, 43% dependency ratio, M:F ratio of 100:105), home of the largest forest in the world	North America, 9.6mil km^2^,~ 329 mil people (~ 75% urban, 51% dependency ratio, M:F ratio of 97:100), world’s largest economy
Social Structure Policies and Social Determinants of Health			
a) Political, economic policies (trade, national & transnational corporations) and civil societies	From a command economy to a developmental state & agricultural-led economy, fair equity and limited influence of corporates; number of civil society organisations & non-governmental organisations (NGOs) increased substantially; strong political commitment	From a military dictatorship with high debit crisis and income inequalities to a stable democracy, fast economic growth with gradual reduction in income inequalities, active participation in civil societies and NGOs; strong political commitment	A neo-liberal economy, high income inequalities among racial/ethnic groups, low political participation as compared to other OECD countries
b) Education, cultural & societal conditions (gender, race, religion)	Free education in public schools, improved enrolment coverage including for females, gross enrolment ratio^d^ is 100.12%, improved women’s household decision making, enhanced women’s economic participation, improved participation of women in high level positions, paid maternity leave	School enrolment increased from 70 to 96% including females’ education, women’s illiteracy declined from 27% in 1980 to 6.5% in 2016, gross enrolment ratio is 109.8%, gaps between white and non-white men/women is getting close, reducing patriarchalism and increasing gender empowerment, reducing gender discrimination, paid maternity leave, achieved significant milestone in gender participation in high positions	Gross enrolment ratio was 99.4%, significant gender, race and SES gaps/inequalities in education coverage, low political participation of minority groups, significant racial and religious discrimination, significant gender discrimination of religion and racial/ethnic minorities, weak economic participation and political decision making in women, no paid maternity leave on a federal level although few private sectors allow paid maternity leave, 40% of women do not qualify for legislated medical and family leave rights
a) Health system policies and indicators	Strong community health programs and network where primary health care is decentralized to the level of kebele (lowest administrative units, ~ 500 households (3500–4000 people), public healthcare expenditure as percentage of GDP is relatively constant (e.g. 4.4% in 2000, 5.5% in 2010, 4% in 2016), improved access to drugs and significant drop in IMR, U5MR & MMR, and universal coverage for HIV treatment (test & treat strategy)	Strong community health programs, access to health services improved through innovative programs, healthcare expenditure as percentage of GDP increased annually (e.g. 6.6% in 2000 to 11.8% in 2016 (45% public)), improved access to drugs and significant drop in IMR, U5MR & MMR, universal coverage for HIV treatment (test & treat strategy)	No universal health coverage, healthcare expenditure as percentage of GDP increased annually (e.g. 12.5% in 2000 to 17.1% in 2016) but sizeable gaps in health coverage, racial disparities in access to health services, IMR, U5MR and MMR decreased in whites but increasing in blacks, MMR increased recently, HIV treatment not free. Some forms of health insurance include mandatory work requirements
i. Primary Health Care	Access to PHC increased through a community health extension program (urban and rural), services decentralized to health centres & health stations, health facilities coverage increased from 76 health posts and 412 health centres to 1600 and 3500	Access to basic health services increased through Family Health Strategy (Doctors, Community Health Workers and other health professionals), Unified Health System (SUS) and “Mais Médicos” programme	Limited access to PHC with strong focus on specialized medicine due to some social insurance system (private market), difficulty in accessing health care, increasing and associated with voting (most with difficulty in accessing health care voted democrat in 2004)
ii. Access to drugs	Health budget increased, established health care finance and community-based health insurance, free maternal health services	Unified Health System provides free access to essential medications, Farmacia Popular provides heavily discounted medications, and Bolsa Família (conditional cash transfers) increased family income.	Limited scope and racial disparities of insurance programs (e.g. Affordable Care Act (ACA)), limited insurance and no free access to drugs, including for HIV treatment
iii. Health indicators	Indicators (1990s to 2017) 1) IMR- 120.2 to 41.0/1000 live births 2) U5MR- 202 to 58.5/1000 live births 3) MMR- 871 to 353/100000 live births 4) HIV Prevalence reduced from 3.2% in 1990 to 0.9% in 2017/8	Indicators (1990s to 2017) 1) IMR – 52.6 to 13.2/1000 livebirths 2) U5MR- 63.1 to 14.8/1000 livebirths 3) MMR- 184 to 58/100000 livebirths 4) HIV prevalence reduced from 3% In 1990 to 0.4% In 2015	Indicators (1990s to 2017) 1) IMR- 9.4 to 5.7/1000 live births 2) U5MR- 11.2 to 6.6/1000 live births 3) MMR- 7.6 to 14/100000 live births 4) HIV prevalence remained 0.34% (850,000 out of 250 mil in 1990 to 1.1 mil out of 323.4 mill in 2016)
b) Agriculture and food supply: Export and localc) consumption	Agriculture as a main source of export, several food security and child nutrition programs as part of SDGs 1&2 (e.g. National Nutrition Program). Overall food security index was 39.4% in 2012 and 36% in 2017 (ranked 100th out of 113 countries).	Main export is agriculture & crude petroleum; established School Food Program, conditional cash transfer program (Bolsa Família); overall food security index was 65.8% in 2012 and 68.4% in 2017 (ranked 39th out of 113 countries).	Main export refined petroleum & cars, planes, helicopters & space craft; Significant gap in food supply compared to the Federal Dietary Guidance; Overall food security index was 85.6% in 2012 and 85% in 2017 (ranked 3rd out of 113 countries).
d) Employment: Conditions of work	Relative rate of unemployment and child labour reduced, paid maternity leave, pension scheme for older people through employer contribution	Increased job stability and wages, reduced unemployment, reduced child labour and slavery, paid maternity leave, pension scheme for older people	Significant underemployment and inequalities relative to other OECD countries, no change of federal minimum wage since 1996, lowest paid sickness leave and public pensions compared to other high-income countries (e.g. Sweden)
e) Income: Wealth & poverty levels	Poverty reduced, purchasing power increased, introduction of social security programs such as health care finance & community-based health insurance, relatively equitable access to resources, Gini coefficient was 0.35 in 2015	Improved social welfare, reduced poverty and inequality, government introduced cash transfer program (Bolsa Familia) and Unified Health System (SUS), Gini coefficient was 0.074 in 2014	Weak social security programs, high rate of income inequalities (Gini index rose by 4%, top 1% of population accounts for 40% of nation’s wealth), relative poverty and child poverty (20% of all children estimated to be living in poverty). Gini coefficient was 0.415 in 2016
f) Housing	Improved housing conditions, access to safe water & ratification of public health regulations	Improved sanitation, access to safe water & housing conditions	Low affordability and inequitable access to housing, significant level of homelessness and housing instability, limited coverage of water supply, and loose public health regulations compared to other developed countries
i. Housing supply	Improved housing supply through ‘Condominium’ (a government loan-based housing program), expanded urbanization to address supply shortage, established Urban Development Package (e.g. Integrated Housing Development Program)	Improved housing supply with adequate electricity, sewage disposal, population living in informal settlements decreased from 37% in 1990 to 22% in 2014	Limited affordable housing compared to other OECD countries, significant cost burden among households; underfunding of the National Housing Trust Fund
ii. Access to safe water	Access to drinking water was 65% (increased by 800%), increases in quantity & reduced distance to collect water	Equitable access to safe water coverage increased to 98%	Inequitable access to safe water in low income and minority families, limited scope of the Healthy Hunger-Free Kids Act program
iii. Effective urban planning & healthy infrastructure	Urban Good Governance Package (promoting effective urban planning, improving infrastructure, justice reform and other packages); WHO Framework Convention on Tobacco Control was ratified in 2014; excise tax bill on alcohol and tobacco approved on November 2019	WHO Framework Convention on Tobacco Control was ratified in 2005	Non-ratification of WHO Framework Convention on Tobacco Control, less regulated markets (e.g. uneven restrictions on advertising of unhealthy products)
i. Environment: sustainable practices & pollution	Ethiopian Environmental Protection Authority established in 1994, Climate resilient green economy strategy including the following proclamations: Environment Impact Assessment Proclamation, Pollution Control Proclamation, Industrial Waste Handling	Strong environmental institutions, creation of Special Secretariat of Environment (SEMA), National Environmental System (SISNAMA), the National Environmental Council (CONAMA) and the Brazilian Institute of Environment and Renewable Natural Resources (IBAMA)	Little action towards a sustainable environment, highest per capita C0_2_ emissions and oil use, some limited environmental protection through Environmental Protection Agency, Clean Air Act, and Clean Water Act

*FDI* foreign direct investments, *GDP* gross domestic product, *HIV* human immune-deficiency virus, *IMR* infant mortality rate, *MMR* maternal mortality rate, *NGOs* non-governmental organisations, *OECD* Organisation for Economic Cooperation and Development, *PHC* primary health care, *PPP* purchasing power parity, *SDGs* sustainable development goals, *U5MR* under-five mortality rate

^a^Calculated from a Preston curve with 2018 GDP per capita, International $ PPP and 2018 life expectancy at birth data, where predicted life expectancy = 5.39 x ln(GDP) + 21.8. GDP = Gross Domestic Product, PPP = Purchasing Power Parity

^b^Life expectancy (LE) relative to GDP status is calculated by subtracting expected LE from actual LE at birth, PAW = Punching Above Weight; PBW = Punching Below Weight

^c^Dependency ratio, an age-population ratio, is the proportion of people who are not in the labour force aged between 0 to 14 and 65+) to people who are in the productive or labour force aged between 15 to 64

^d^Gross enrolment ratio (GER) is the number of students enrolled in a given level of education regardless of age divided by the population of the age group which officially corresponds to the given level of education, and multiplied by 100. A high GER generally indicates a high degree of participation. A GER value approaching or exceeding 100% indicates that a country is, in principle, able to accommodate all of its school-age population

Our next step was to produce a narrative synthesis of the different sources of information for each country. The results are described below.

## Ethiopia

Ethiopia is one of a handful of African countries that has shown life expectancy gains since 2000 despite the country having a low per capita GDP [16]. Life expectancy in Ethiopia has increased from 38 in the 1960s, to 49 by 1995 and to 66 in 2018. In 2018 Ethiopia punched above its economic weight by 3.03 years. The socioeconomic gradient in life expectancy has also flattened [17]. Several key health indicators have improved in the last two decades, e.g. Ethiopia achieved Millennium Development Goal 4 on under 5 mortality 3 years before the deadline [18, 19]; between 1995 and 2015 the infant mortality rate (IMR) reduced from 105 to 51 deaths per 1000 livebirths, and the maternal mortality ratio (MMR) reduced from 1080 to 353 deaths per 100,000 births [20]. HIV incidence and AIDS related deaths have declined by over 80% since 1990s [21]. HIV prevalence decreased from 3.2% in 1990 to 0.9% in 2017. These health improvements are likely to have contributed to Ethiopia’s life expectancy gain. Plausible reasons for these achievements are discussed below.

### Political economic and institutional context and history

Ethiopia has been ruled by monarchic (before 1970s) and autocratic (up to 1990) governments. Under these regimes, the country was characterized by an unstable political economy, a high burden of preventable infectious diseases, and limited and inequitable access to health, education and infrastructure [22]. Post-1990, the Ethiopian People’s Revolutionary Democratic Front party ruled the country with a ‘revolutionary democratic’ (later ‘developmental democratic’) ideology. Since then, Ethiopia has enjoyed relative peace, and increasing access to and equity in key social determinants such as education and primary health care [23]. National and transnational trade agreements and foreign direct investments in Ethiopia have increased, and have targeted equitable infrastructure such as health facilities and farmer training centres. Under the leadership of late prime minister Meles Zenawi, the country resisted the privatization and neoliberalism promoted by international agencies such as the World Bank [24, 25]. Neoliberalism is an economic theory developed in the 1940s and 1960s that has gained prominence since the 1980s, prominently championed by Ronald Reagan in the United States and Margaret Thatcher in the United Kingdom. It emphasises reducing government spending and intervention and promotes free markets, free trade, property rights, and the sovereignty of individuals [26, 27].

### Social determinants of health

#### Education and gender

Primary and secondary education is free in public schools. The literacy rate in the country improved from 27% in 1994 to 49% in 2015 [28], and the gross enrolment ratio (GER) has been increasing and was 100.12% in 2017 [29]. In particular, women’s education in Ethiopia has improved in the last decade. Females’ national gross enrolment ratio increased from 26% in 1997 to 97% in 2015 [30, 31]. Ethiopia has been working to improve gender empowerment in decision making in the domestic sphere, expanding women’s economic opportunities, and their participation in political decision making, including recruitment to senior positions— the president, 50% of ministers, and 39% of the parliament were women in 2018/9: among the highest rates in Africa next to Zimbabwe (44%), Rwanda (39%) and South Africa (39%) [32].

#### Health system and infrastructure

The health policy of the federal government differs from that of previous governments primarily through incorporating elements of decentralization, democratization, promotion of participation of the private sector and NGOs. It has included a focus on inter-sectoral collaboration, collaboration with neighboring countries on health care, and regional approaches to reduce transmission of infectious diseases and other health threats [33].

Evidence indicates that access to health care plays an important role in population health and that countries with universal health coverage fare better than those without [34]. Previously, high out-of-pocket costs had reduced health care access in Ethiopia [35]. To alleviate this, the government established a health care financing (HCF) strategy in 1998 [36, 37] which included a fee-waiver for people who could not pay for health services. Community based health insurance (CBHI) [35, 38] was established in 2011. Together, these programs have improved the quality, equity and accessibility of health services and contributed to higher life expectancy [36, 38]. However, inequalities in healthcare resources persist. For example, a study conducted in 2019 revealed high inequities in the distribution of doctors, nurses, midwives, and health officers across Ethiopia [39]. While over 80% of the population in Ethiopia live in rural areas, approximately 90% of hospitals are in urban areas [40].

Ethiopia aims to achieve universal health coverage through the provision of primary health care (PHC) services [23]. PHC has been decentralized to kebele level: the lowest administrative units in Ethiopia, with an average of 500 households and five health posts that serve 3000 to 5000 people each. Kebeles share a health centre that services 25,000–40,000 people and coordinates the health posts [41]. The number of health posts and health centres has increased from 76 and 412 in the early 1990s to 1600 and 3500 in 2015 respectively [42, 43]. One core component of PHC in Ethiopia has been the Health Extension Program that provides routine house-to-house visits to deliver four principal strategies (disease prevention and control, family health services, hygiene and environmental sanitation, and health education and communication) by deploying community-based health workers (mostly women), called Health Extension Workers (HEWs), who are given 1 year of training [44, 45].

Ethiopia has also been training and deploying volunteer community health workers, women’s development armies, and traditional birth attendants. These and the HEWs have expanded immunization, family planning, antenatal and HIV testing, improved awareness of health, health service utilization, and reduced home delivery, and subsequently contributed to the reduction of infant and maternal mortality [45, 46]. Furthermore, the HEWs have contributed to improving sanitation coverage, treating respiratory tract infections and diarrhoeal diseases [47], the major causes of years of life lost in the 1990s in Ethiopia [48]. The Health Development Army comprises over 3 million volunteers (predominantly women) who engage in multi-purpose health promotion activities including community empowerment and disease prevention [49]. These volunteers are trained and supervised by the HEWs, and are organized to provide services by neighborhood.

Ethiopia’s Emergency Obstetric and Newborn Care program, launched in 2008, has been estimated to have reduced infant mortality and averted 60% of maternal deaths [50]. The decline in HIV mortality which has resulted from *test and treat* strategies and other community-based HIV care interventions is likely to have contributed to increased life expectancy. As described above, the establishment of HCF and CBHI improved access to medication.

Access to safe water has improved. The overall coverage of access to drinking water in 2016 was 65%, an 800% increase compared to the 1990s. In 2015, Ethiopia met the Millennium Development Goal 7c target on access to drinking water supply [51]. A study in central Ethiopia [52] found that most water schemes were located at a distance of less than 2 km with less than a 30 min round trip to fetch the water. However, there were still challenges: queues could be long, and only 15% of people received 20 l of water per day per capita.

Ethiopia has implemented healthy urban planning, infrastructure policy, and public health regulations. For example, the government established an Urban Good Governance Package that focuses on promoting effective urban planning, improving infrastructure, justice reform and other initiatives [53]. Ethiopia ratified the WHO Framework Convention on Tobacco Control in 2014 [54]. Furthermore, the excise tax bill on alcohol and tobacco was approved in 2019 [55].

#### Income, wealth and poverty

Ethiopia’s economy depends on agriculture, accounting for 40% of GDP, 80% of exports and 75% of the country’s workforce. Ethiopia’s unemployment has reduced from 2.9% in 1991 to 1.8% in 2018 [56]. Sick and maternity leave is paid leave in all public institutions and most private organisations (excluding informal sector workers) [57]. There is a nationally allotted pension scheme for older people through employer contributions for both public and private organisations. The Gini coefficient (a measure of income inequality [58]) in Ethiopia has decreased from 0.44 in 1995 to 0.35 in 2015 (lower is more equitable) [59].

Ethiopia has implemented food security and nutrition programs such as the Productive Safety Net Program, and *Seqota* declaration (a multi-sectoral collaboration plan of 11 ministries) to end child undernutrition by 2030 [60]. The *Seqota* declaration is managed under the National Nutrition Program [61], an initiative established in 2013 to improve the nutritional status of women, adolescents, young children and infants [62]. The program has helped reduce the prevalence of stunting and other malnutrition problems [63].

#### Housing

Housing conditions in Ethiopia have improved over time. Ethiopia has introduced a subsidized and government loan-based housing program called ‘Condominium’, providing accommodation for low- and middle-income people who are able to pay the total cost over 20 years [64]. Urbanization has increased in Ethiopia, and the government has established an integrated housing development program to provide mass housing to residents in urban informal settlements [53].

#### Environment

Ethiopia is addressing sustainability and environmental protection. The Environmental Protection Authority was established in 1994. Ethiopia has launched the Climate-Resilient Green Economy (CRGE) initiative, and made several proclamations that support a green economy and clean environment, e.g. on Environment Impact Assessments, Pollution Control, and Industrial Waste Handling.

### Civil society organisation and political participation

The first formal civil society group in Ethiopia was established in 1930, and the first international and local NGOs began operating in 1960 [65]. Civil society was oppressed under the feudal monarchy. When armed forces overthrew emperor Haile-Selassie, civil societies were again co-opted and barred [65]. The Mengistu regime ruthlessly suppressed national NGOs, media, professional associations, the business sector, trade unions, academia and other sectors of civil society [65].

In 1991 when the Mengistu regime was defeated and a transitional government was established, civil society began to be re-established [65]. The number of civil society organisations increased substantially (e.g. 310 NGOs were officially registered in 1999, although they were concentrated in the capital city) and have contributed to the political and economic revitalization of the nation. Small business grew swiftly, the media gained credibility slowly, and professional associations were again established, albeit gradually. In 2014, there were 3077 registered civil society organisations, delivering more than 2600 projects with a total budget of US$1.8 billion (Birr 35.8 billion) [66].

## Brazil

Brazil punched 2.01 years above its predicted life expectancy for its GDP in 2018. Life expectancy in Brazil has increased from 54 in the 1960s, to 68.5 in 1995 and 76.3 (72.8 years for men, 79.9 years for women) in 2018 [67]. Between 1990 and 2015 infant mortality fell from 47.1 to 13.5 per 1000 live births, under five mortality declined from 53.7 to 15.6 per 1000 live births, and maternal mortality reduced from 143.2 to 59.7 per 100,000 live births [68]. Health equity has increased: the difference in under five mortality rates between the top and bottom wealth quintiles has decreased from 65 deaths per 1000 children in 1991 to 31 deaths per 1000 in 2001–2002 [69]. Possible drivers of these outcomes are discussed below.

### Political and institutional context and history

Brazil has moved (since 1985) from a military dictatorship with an unstable political economy, where democratic institutions and procedures were compromised, and severe human rights violations were recorded, to a stable democracy marked by pluralization and a presidential system [70]. In the 1980s Brazil experienced hyperinflation and a serious foreign debt crisis with economic stagnation and recession, and concentration of income among a small percentage of the population. Inflation was contained in the 1990s, with low economic growth during that time. After 2000 Brazil had more substantial but still modest economic growth [70].

### Social determinants of health

#### Education and gender

Brazil has undertaken several large-scale social reforms that have resulted in increases in school attendance and literacy rates [71]. The overall gross enrolment ratio was 115.5 in 2017, and females’ primary school gross enrolment ratio was 113.4 in 2017 [29]. The female illiteracy rate in Brazil has dropped from 27.7% in 1980 to 6.5% in 2016, and the proportion of women with basic education (> 8 years) increased from 12.9% in 1981 [72] to 64% in 2016 [73]. It is estimated that for each 10% increase in female literacy in Brazil there was a 16.8% reduction in infant mortality [74].

Brazil has the world’s largest affirmative action program for university entrance and in 2012 a national law was enacted reserving places in all federal public universities through a quota system based on both socioeconomic and racial inequalities (although its implementation has been criticised) [75]. Brazil has achieved significant milestones in gender equality in participation in senior/leadership positions particularly in science, technology and innovation [76].

#### Health system and infrastructure

Urbanization increased from 67.6% of the population living in cities in 1980 to 84.4% in 2010, and this has been associated with improved access to health care services, safe and accessible drinking water, and other basic infrastructure including electricity [77].

Brazil increased access to health care through the establishment of the Unified Health System (SUS) [71, 78] in 1988, the Community Health Worker Programme [79] in 1991 and the Family Health Strategy (ESF) [80] in 1994. By 2015 the family health strategy (ESF) covered 60.6% of the population [81]. In places where ESF coverage was greater than 80%, the infant mortality in the poorest quintile was 1.5 times greater than in the richest quintile of municipalities. In municipalities with ESF coverage between 60 and 80%, this increased to 1.8 times greater inequalities; and in municipalities with less than 60% ESF coverage, this increased to 2.6 times greater inequalities [69]. It has been estimated that for each 10% increase in coverage of the ESF, there was a 4.6% reduction in infant mortality [74].

The 1988 Brazilian Constitution established a universal health system that is free-of-charge for users and launched major investments in training of health professionals, production of pharmaceuticals, public health surveillance and health research and technology development [71]. The 2013 “Mais Médicos” programme (More Doctors Programme), further expanded access to health care, encouraging doctors, including foreign doctors, to go to underserved remote areas [82].

Maternal and child programs have been implemented, including mass vaccination campaigns, a national program for the reduction of infant mortality, and a national women’s health program [83]. Since 1981, Brazil has had a National Programme for the Promotion of Breastfeeding involving policy makers, health workers, mass media, and civil society organisations [84]. Breastfeeding duration increased from 2.5 months in the 1970s to 14 months in 2006; and exclusive breastfeeding in children under 6 months increased from 3.6% in 1986 to 37.1% in 2006 [85]. In São Paulo, it was estimated that the impact of this increased breastfeeding reduced preventable deaths of children in the first year of life by 9.3% in the period of 1999–2000 [86].

There has been a significant reduction in stunting rates in the last 30 years (from 37.1% in 1974–75 to 7.1% in 2006) and a reduction of socioeconomic inequalities in stunting. In 1989, children from families in the lowest wealth quintile were 7.7 times more likely to have stunted growth than those from families in the highest wealth quintile. This ratio dropped to 6.6 in 1998, and 2.6 in 2007 [69]. Prevalence of overweight and obesity is increasing in Brazil, linked to increased consumption of ultra-processed foods [87].

The proportion of households with access to adequate sanitation has increased significantly (from 27.7% in 1980 to 54.9% in 2011), but almost half of homes still do not have an adequate sewage system [80]. The proportion of households with access to safe water supply increased from 54.9% in 1980 to 84.7% in 2016 [80]. For each increase of 10% in coverage of access to safe water supply, a 2.9% reduction in infant mortality has been observed [80].

#### Income, wealth and poverty

There has been a gradual reduction in income inequalities in Brazil and a 70% reduction in the number of people living in absolute poverty: from 24.7% in 2001 to 7.4% in 2014 [68]. Regional differences and differences between the richest and poorest have decreased, reflected in an improvement in the Gini coefficient from 0.635 in 1989 to 0.533 in 2017 [88]. Brazil’s economy changed from a focus on mining and agriculture to manufacturing and service industries. There was also a real rise in the minimum wage and employment rate in the mid 2000s [89]. The Brazilian government has introduced several policies to reduce child labour and slavery, increase job stability and wages, reduce unemployment, narrow inequalities in income, and improve food security [89, 90]. For example, the Cardoso government established the Child Labor Eradication Program (PETI) to fight against child labour, a program that contributed to reducing the child labour rate from 13.7 to 8.2% between 1995 and 2002 [90].

The establishment of the Brazilian Ministry of Social Development in 2004 led to a substantial increase in the social protection budget, including the Bolsa Familia program. Launched in 2003, Bolsa Família [91] is one of the largest conditional cash transfer programs in the world, with 13.8 million families covered in 2015. It consists of a monthly transfer of US$18 to US$175 for poor families with children, adolescents or pregnant women when they comply with conditions related to health and early childhood education [91].

Brazil has additional social security schemes that are likely to promote good health and wellbeing. Social pension schemes that support people aged 65 years and over cover more than 86% of this age group, which is among the highest in the Latin American region. The Continued Benefit of Social Assistance payment scheme has also provided support for poor older individuals and people with a disability [92].

Brazil provides a minimum of 120 days paid maternity leave at the federal level. National programs targeting food security and nutrition were introduced in 2000 [93]. The expanded School Feeding Program is one example, reaching over 45 million students [94]. The program contributes to the development and educational achievement of students through meeting their nutritional needs (using local family farming) while in the classroom [94].

#### Housing

Housing conditions have improved with increasing urbanisation. The federal government, in partnership with local governments, has implemented several programs supporting residents of informal settlements over the last 20 years. The proportion of the population living in informal settlements has decreased from 37% in 1990 to 22% in 2014 [95]. This has been accompanied by an increase in housing supply with adequate electricity.

#### Environment

Despite Brazil’s economy changing from agriculture to manufacturing, under governments prior to that of President Bolsonaro, the country has sought to improve environmental sustainability. Several initiatives and programs have been launched to maintain biodiversity and a clean environment, including the creation of Permanent Protection Areas [96] to surround rivers, and the establishment of the Brazilian Institute of Environment and Renewable Natural Resources, the Special Secretariat of Environment, the National Environmental System, the National Environmental Council, and the Brazilian Institute of Environment and Renewable Natural Resources.

### Civil society and political participation

In the mid-1980s during the democratic transition period, there was a rapid increase in civil society organisations in Brazil. In the 1980s, a Catholic church-based NGO run by volunteers, Pastorate of the Child, was very active at a time when health care had very low coverage, especially in less privileged areas. In the 1990s, Brazil experienced a popular movement against hunger. From 2003 onwards, when a representative of the Workers’ Party took over the presidency, there was an intensification of public participation in the formulation and monitoring of the public policies that occurred via committees, councils and conferences. This was accompanied by new voluntary associations, new practices and institutions for democracy and policy making. Civil participation in Brazil has been argued to have been an integral part of health system governance and to have contributed to health care reforms [71].

## United States of America

The United States of America is a notable case of a high-income country that has life expectancy lower than would be expected given its high gross domestic product (− 2.87 years). Our analysis suggests that is a result of the impact of a broad socio-political and economic context unsupportive of health, and several adverse social determinants of health factors [97, 98].

### Political and institutional context and history

The election of the Republican president Ronald Reagan in 1980 led to a rapid and intensive implementation of neoliberalism in the US [99]. Successive governments implemented economic deregulation including increased labour market flexibility (e.g. restrictions on trade union and employment rights), reductions in social expenditure and public services (e.g. a 1996 act imposed a lifetime limit of 5 years for the receipt of welfare benefits) [97], the pursuit of low inflation (at the cost of higher rates of unemployment) [100], and lower taxation rates particularly for corporations and more wealthy individuals. These neoliberal reforms resulted in large increases in income inequality; a smaller social safety net; and higher rates of poverty and unemployment. These economic factors all have clear implications for why the United States punches below its economic weight in terms of health. In terms of income inequality, Wilkinson and Pickett [101] have established that countries with higher levels of income inequality have worse health outcomes – higher IMR, lower life expectancy, higher rates of obesity, excess risk of premature mortality, increased homicide rates and higher levels of mental ill health [101].

### Social determinants of health

#### Education and gender

Women in the United States are affected by discriminatory policies, and inequities in health care access and cost, more than in other comparable Organisation for Economic Co-operation and Development (OECD) countries [102]. Women in the United States also have weak economic participation compared to women in other OECD countries [103]. The United States is the only high-income country that offers no paid maternity leave at the federal level, although there are a few private organisations who offer it [104]. Unpaid but protected maternity leave has been legislated by the federal government, but approximately 40% of women do not qualify [105]. Only 12% of women in the private sector access paid maternity leave [105]. A quarter of the lower house in the United States is made up of women, reflecting inequitable participation of women in high level political decision making. This proportion is lower than in other high income countries, such as Australia (49%), Canada (47%) and Belgium (47%) [32]. The United States has a gross education enrolment ratio of 99.4% but education coverage shows significant disparities by gender, race and socio-economic status [29].

#### Health system and infrastructure

Health in the United States constitution is the responsibility of states, not the federal government, which leads to large diversity in states’ public health spending, regulation, and performance [106].

The United States has relatively worse population health behaviours than other comparable countries. The country has the highest average calorie intake in the world, with high fat and sugar foods contributing [107]. Regulating the formulation of unhealthy products (e.g. by limiting levels of saturated fat, salt and refined sugar in food and drinks), their availability, marketing, and price are effective means of reducing their consumption and impact [108–110]. Yet the United States remains one of the least regulated food markets among high income countries, and is, for example, one of only a small number of high income countries not to have ratified the Framework Convention on Tobacco Control [111]. Disease prevention policies (especially in terms of alcohol and tobacco control) are less extensive in the United States compared to Europe [112]. Increased access to unhealthy products not only increases the overall burden of non-communicable diseases in the United States but also directly contributes to inequalities in these diseases since less advantaged population groups (e.g. those experiencing poverty) are more likely to experience associated harms, and are less likely to benefit from public health interventions [113]. Although 99.3% of the population in the US used at least basic drinking water services in 2017 [114], the statistics among minority and low-income populations are lower (e.g. 11% of Indigenous communities do not have safe piped water) [115].

The United States spends the most in the world on health care - in absolute terms, per head of population and as a proportion of national income (around 18% of United States GDP), but it does not have universal health coverage [116] and a large proportion of the high expenditure is spent on funding and regulating a health care market – rather than patient care [100]. Unlike other wealthy countries that operate a social insurance system (whereby the government, employers and employees co-fund health care via regular set contributions e.g. France and Germany) or a national health system (where health care is funded by the government based on general taxation e.g. the UK, Australia, Sweden or New Zealand), the United States system is a complex hybrid. There are national health insurance programs (Medicare for those aged 65 and over, The Child Health Insurance Program for children), National Health Services for some population (the Veterans Health Administration and the Indian Health Service), employer-sponsored health insurance, and completely private insurance options that are paid 100% out of pocket. There are also government funded schemes for the very poor and disabled (Medicaid), which have different levels of eligibility and coverage depending on the state [117]. Although over 50% of all healthcare expenditures in the United States are public, these fragmented schemes generally do not provide comparable levels of coverage or financial protection as those in other high-income countries. The most recent set of health reforms, ‘Obamacare’ (the Patient Protection and Affordable Care Act of 2010) increased access to health insurance, but around 10% or 33 million Americans remain without any health insurance and therefore only have access to emergency care or services provided through a poorly-funded set of community health centres. These “safety-net” providers may not provide sufficient access to prevention or primary or secondary care and may also levy charges on users [118]. Millions of others remain “under-insured” whereby their health care policies do not cover the full range of health services [116]. Furthermore, the high cost of health care causes poverty, loss of mortgages and homelessness [119].

#### Income, wealth, and poverty

The United States’ main exports depend on refined petroleum and cars, planes, helicopters, and spacecraft. The nation has higher poverty rates and income inequality compared to most high-income countries. For example, children in the United States are more likely to be raised in poverty than children in peer countries, and over 17% of United States citizens experience ‘relative poverty’ (defined as having less than 50% of the average [median] national income) compared to 11% in the United Kingdom and around 7% in Denmark [107]. The United States has less social security to help protect against the consequences of adverse economic and social conditions than in other countries. A comparative analysis of the association between welfare benefit generosity and life expectancy rates from 1970 to 2011 found that life expectancy in the United States would be almost 4 years longer, if it had the average social policy generosity of other high income nations [120]. The United States also provides the lowest level of unemployment, sickness and public pensions compared to other high income countries [120]. Research has also demonstrated that countries with more generous public social security systems have lower IMR, lower overall mortality rates, less mortality at younger ages and, albeit to a lesser extent, increased life expectancy at birth [121, 122].

Growing income inequalities is demonstrated by a 4% rise in the Gini index between 1995 and 2005. In 2016, the Gini coefficient of the US was 0.415 [88]. The top 1% of the population now accounts for 40% of the nation’s wealth, which makes the United States the most unequal of high income countries [123]. In 2006, full-time black male and female workers earned 80 and 84%, respectively, of white men and women’s earnings. The Federal minimum wage has not changed since 1996.

#### Housing

The United States has shortfalls in affordable housing supply, indicated by the approximately 18.5 million very low-income households eligible for federal rental housing assistance (this number having increased by 3.8 million from 1993 to 2013) [124]. The National Housing Trust Fund, the housing resources targeted at building, preserving, rehabilitating, and operating rental housing for very low-income people, has been consistently underfunded [124, 125].

#### Environment

The United States has the highest per capita rate of CO_2_ emissions and oil use in the world and yet has not adopted sustainable environmental policies to any significant degree [126].

### Civil society organisation and political participation

In terms of political participation, the United States ranks amongst the lowest of wealthy countries for voting participation rates and levels of trust [127]. It also has the lowest rate of trade union membership amongst wealthy countries – restricting the representation of working-class interests in policy and politics. Only 12% of the United States workforce is a member of a trade union compared to 68% in Sweden [128]. Research has shown that the political incorporation of minority groups is robustly associated with better health amongst those groups [129, 130]. The United States was a historical laggard in terms of the incorporation of minority groups – with formal equal civil rights for African Americans only achieved in the 1960s. An example of the effects of such political incorporation on health comes from a study of the abolition of ‘Jim Crow laws’ in the Southern states of the United States such as Mississippi as a result of the 1964 Civil Rights Act [129]. ‘Jim Crow laws’ were a legalised form of racial discrimination and segregation – and were similar to the apartheid regime in South Africa [129]. The study found that from 1960 to 1964, the African American IMR was 20% higher in the Jim Crow states than in the non-Jim Crow ones, whereas after abolition (and the increased political emancipation of African Americans in these states), the gap disappeared [130]. These gains were made in large part because of an active civil rights campaign.

## Discussion

The results of scoping review of the three countries presented here shine a light on possible political, structural, and social determinants that are driving global inequities in life expectancy, and help explain why some countries punch above or below their economic weight. Examination of punching above or below weight outcomes for our three case study countries indicated that they include two consistently above weight countries (Ethiopia and Brazil), and one consistently below weight country (the United States). Our reviews reinforce findings from earlier research that investment in PHC, maternal and child health, and universal health coverage have a strong role to play in achieving positive and equitable population health outcomes - in all countries regardless of their wealth. Universal health coverage is one of the Sustainable Development Goals, although there is concern that the way it is framed invites privatisation and more selective approaches to PHC compared to previous World Health Organization approaches, e.g. the Alma Ata Declaration [131]. Comprehensive PHC approaches are likely to positively contribute to population health, as evidenced by the gains in positive health outcomes from the very strong community-based PHC and health promotion in Ethiopia and Brazil and its relative absence in the United States.

However, our findings show that the role of drivers outside of the health system – social, commercial and political determinants – are also important. The weak welfare provisions in the United States are implicated in its poor population health performance. The social security and affordable housing initiatives in Brazil and Ethiopia are likely to play an important role in the life expectancy gains experienced by these two countries in the recent past. Similarly, the strength and growth of civil society in recent decades in Ethiopia and Brazil contrasts with the poor unionisation and low political participation in the United States. A strong civil society has been associated with more redistributive policies in a country [132, 133], however civil society’s role in contributing to positive and equitable population health outcomes has received less research attention.

More unequal societies have worse health, and other social outcomes [101]. It is not just the mathematics of inequities pulling down the overall average health outcomes, but also that an unequal society has negative effects on all groups [101]. Wilkinson and Pickett propose inequities affect health through interpersonal pathways such as dissatisfaction with one’s own social status, lack of social cohesion, and trust [101]. The two punching above weight countries had both sought to act on inequities, while the punching below weight United States has large socioeconomic and racial inequities. The breadth of possible policies and social determinants of health driving population health outcomes were on display in all three countries. Rather than acting as a list of discreet drivers, the policies and social determinants of health we identified can be expected to interact to produce population health outcomes. For example, in the United States, shortcomings in affordable housing, poor working conditions, and a weak social security system are likely to combine to exacerbate poverty and ill health. It is also important to note that these dynamics and specific determinants will change over time. For example, in Ethiopia, neoliberal approaches to public policy are encroaching, as demonstrated by recently approved legislation enabling the privatization of public services [55, 134]. The election of Bolsonaro in Brazil is heralding policy directions less supportive of population health [135, 136]. In the United States, life expectancy is falling [137], and the policies of the current administration that are not supportive of population health may contribute to a further decline [138–142]. For example, the repealing of the Affordable Care Act would lead to a potential loss of insurance coverage for millions of Americans [138, 143].

We sought to examine how much we could answer our research question on drivers of punching above or below weight status of different countries using existing literature and data sources. While the findings clearly highlight the importance of studying a country’s policy context, there were limitations to what we could achieve with this approach. One clear remaining question is how to ascertain attribution of population health outcomes, and how best to tease out the role of different policy approaches and sectors. One possible approach would be to continue this work by building a database of political, environmental, and social determinants with a greater number of countries in the punching above or below weight categories, that could be interrogated to look for relationships between policies, social determinants, and population health outcomes. There were instances where timelines of available data differed slightly between countries, e.g. for HIV, we used 2017 data in Ethiopia, 2015 in Brazil and 2016 in the United States. While we did not seek to directly compare the countries, this shows the challenges in establishing good quality, comparable data.

Within-country equity is a vital consideration, as well as comparing the overall population health achievements between countries. Within-country inequities were identified in all three countries. For example, in Ethiopia, females live 2 years longer than males [144]. In Brazil, people living in the most affluent regions live 5 years longer than those living in the less wealthy regions [145]. In the US, white Americans live 7 years longer than African Americans [146]. The gap in life expectancy at birth between the top 1% and bottom 1% of individuals in the United States was over 10 years (14.6 years for men and 10.1 years for women) [147], and there is a six-year life expectancy gap between the states with the highest life expectancy (Hawaii), and the lowest (Mississippi) [147]. We found within-country equity data was not always as readily available or comparable as general life expectancy data.

Our scoping reviews aimed to be comprehensive of the possible sectors and social determinants of health that may have affected life expectancy outcomes relative to national income, following the framework in Fig. 1. In seeking to answer our research question, we have focused our analysis and reporting on understanding the positive factors that may have contributed to Ethiopia and Brazil punching above their weight and the negative factors that may have contributed to the United States punching below weight. We acknowledge there are also negative factors in Ethiopia and Brazil, and positive factors in the United States that we have omitted for parsimony. The United States is also being held to a higher standard as a wealthier country than Ethiopia and Brazil in our determinations. The two positive countries should not be seen as simple hagiographies, but an attempt to isolate the positive factors contributing to their punching above weight. We have also seen how the prospects for health can change rapidly with recent political and economic changes in both Ethiopia and Brazil.

We focused on the national level. However, there are global factors driving countries’ population health outcomes, including climate change, the behaviour of transnational corporations, and global trade structures. Climate change is emerging as a critical determinant of population health that will affect countries differentially, and will affect populations within countries differently, likely in ways that exacerbate existing inequities [148]. It is of note that per capita energy use, oil use, and carbon dioxide emissions are far higher in the United States, our punching below weight country, than for every other country in the world [149].

## Conclusions

While the reliance on existing literature and data inevitably left some questions unanswered, particularly in regard to how to tease out attribution, our analysis is valuable in highlighting the range and importance of social determinants of health, not just health systems, to understand why individual countries punch above or below their weight in life expectancy relative to their wealth. These drivers are complex, interdependent, and constantly changing, and global forces including climate change, transnational corporations, and trade patterns, are also critical determinants. Finally, our review emphasised the importance of considering the equity of the distribution of population health within countries, as well as overall population health outcomes. Further research identifying clear policy drivers in a wider range of countries is needed so that all countries can achieve the highest possible level of equitable population health.

## Supplementary Information

**Additional file 1: S1 doc**. Table 2: Punching above or below weight status and selected context and social determinants of health indicators of Ethiopia, Brazil and the United States of America, 1990s-2018 (with citations)

## Data Availability

The dataset supporting the conclusions of this article is available at https://data.worldbank.org/indicator/SP.DYN.LE00.IN for life expectancy, and https://data.worldbank.org/indicator/NY.GDP.PCAP.CD for gross domestic product.
