# Supplementary material for: Why do some countries do better or worse in life expectancy relative to income? An analysis of Brazil, Ethiopia, and the United States of America
Source: Int J Equity Health. 2020 Nov 10;19:202. doi: 10.1186/s12939-020-01315-z (PMC7654592; doi:10.1186/s12939-020-01315-z)
Supplement: Supplementary file 1 — Additional file 1: S1 doc. Table 2: Punching above or below weight status and selected context and social determinants of health indicators of Ethiopia, Brazil and the United States of America, 1990s-2018 (with citations) [file 12939_2020_1315_MOESM1_ESM.docx]

Table 1: Punching above or below weight status and selected context and social determinants of health indicators of Ethiopia, Brazil and the United States of America, 1990s-2017 (with citations)

| Characteristics | Ethiopia | Brazil | USA |  |
| --- | --- | --- | --- | --- |
| Life expectancy at birth, 1997 | 50.3[1] | 68.6[2] | 76.4[3] |  |
| Actual life expectancy at birth, 2018 | 66.2[1] | 75.7[2] | 78.5[3] |  |
| Expected life expectancy at birth^[[1]](#footnote-1)^, 2018 | 63.2[4] | 73.7[4] | 81.4[4] |  |
| GDP per Capita PPP, 2018 | $2,153[5] | $14,941[5] | $62,840[5] |  |
| Life expectancy relative to GDP (PAW or PBW) status^[[2]](#footnote-2)^ | PAW, +3.0 years | PAW, +2.0 years | PBW, -2.9 years |  |
| Context | | | | |
| Country geography and population demographics | East Africa, 1.2mil km^2^, 100mil people (~80% are rural, 82% dependency ratio^[[3]](#footnote-3)^, male: female (M: F) ratio is 100:101), land locked [6] | Latin America, 8.5mil km^2^ ,~210 mil people (~80% urban, 43% dependency ratio, M:F ratio of 100:105), home of the largest forest in the world[6] | North America, 9.6mil km^2^ ,~329 mil people (~75% urban, 51% dependency ratio, M:F ratio of 97:100), world’s largest economy [6] |  |
| Social Structure Policies and Social Determinants of Health | | | |  |
| 1. Political, economic policies (trade, national & transnational corporations) and civil societies | From a command economy to a developmental state & agricultural-led economy, fair equity and limited influence of corporates; number of civil society organizations & non-governmental organizations (NGOs) increased substantially; strong political commitment[7-9] | From a military dictatorship with high debit crisis and income inequalities to a stable democracy, fast economic growth with gradual reduction in income inequalities, active participation in civil societies and NGOs; strong political commitment[10] | A neo-liberal economy, high income inequalities among racial/ethnic groups, low political participation as compared to other OECD countries[11-13] |  |
| 1. Education, cultural & societal conditions (gender, race, religion) | Free education in public schools, improved enrolment coverage including for females, gross enrolment ratio^[[4]](#footnote-4)^ is 100.12%, improved women’s household decision making, enhanced women’s economic participation, improved participation of women in high level positions, paid maternity leave[12, 14-16] | School enrolment increased from 70% to 96% including females’ education, women’s illiteracy declined from 27% in 1980 to 6.5% in 2016, gross enrolment ratio is 109.8%, gaps between white and non-white men/women is getting close, reducing patriarchalism and increasing gender empowerment, reducing gender discrimination, paid maternity leave, achieved significant milestone in gender participation in high positions[12, 17-19] | Gross enrolment ratio was 99.4%, significant gender, race and SES gaps/inequalities in education coverage, low political participation of minority groups, significant racial and religious discrimination, significant gender discrimination of religion and racial/ethnic minorities, weak economic participation and political decision making in women, no paid maternity leave on a federal level although few private sectors allow paid maternity leave, 40% of women do not qualify for legislated medical and family leave rights [12, 20, 21] |  |
| 1. Health system policies and indicators | Strong community health programs and network where primary health care is decentralized to the level of kebele (lowest administrative units ~ 500 households (3500-4000 people), public healthcare expenditure as percentage of GDP is relatively constant (e.g. 4.4% in 2000, 5.5% in 2010, 4% in 2016), improved access to drugs and significant drop in IMR, U5MR & MMR, and universal coverage for HIV treatment (test & treat strategy) [22-28] | Strong community health programs, access to health services improved through innovative programs, healthcare expenditure as percentage of GDP increased annually (e.g. 6.6% in 2000 to 11.8% in 2016 (45% public)), improved access to drugs and significant drop in IMR, U5MR & MMR, universal coverage for HIV treatment (test & treat strategy) [17, 29-32] | No universal health coverage, healthcare expenditure as percentage of GDP increased annually (e.g. 12.5% in 2000 to 17.1% in 2016) but sizeable gaps in health coverage, racial disparities in access to health services, IMR, U5MR and MMR decreased in whites but increasing in blacks, MMR increased recently, HIV treatment not free. Some forms of health insurance include mandatory work requirements[33, 34]. |  |
| - 1. Primary Health Care | Access to PHC increased through a community health extension program (urban and rural), services decentralized to health centres & health stations, health facilities coverage increased from 76 health posts and 412 health centres to 1600 and 3500[22-24, 35, 36] | Access to basic health services increased through Family Health Strategy (Doctors, Community Health Workers and other health professionals), Unified Health System (SUS) and “Mais Médicos” programme[17, 29, 32] | Limited access to PHC with strong focus on specialized medicine due to some social insurance system (private market), difficulty in accessing health care, increasing and associated with voting (most with difficulty in accessing health care voted democrat in 2004)[33, 34, 37, 38]. |  |
| - 1. Access to drugs | Health budget increased, established health care finance and community-based health insurance, free maternal health services[39-42] | Unified Health System provides free access to essential medications, Farmacia Popular provides heavily discounted medications, and Bolsa Família (conditional cash transfers) [17, 29, 43] increased family income. | Limited scope and racial disparities of insurance programs (e.g. Affordable Care Act (ACA)), limited insurance and no free access to drugs including for HIV treatment[44] |  |
| - 1. Health indicators | Indicators (1990s to 2017)   - - 1. IMR- 120.2 to 41.0/1000 live births[27]     2. U5MR- 202 to 58.5/1000 live births[25, 26]     3. MMR- 871 to 353/100000 live births[27]     4. HIV Prevalence reduced from 3.2% in late 1990s to 0.9% in 2017/8[28] | Indicators (1990s to 2017)   - - 1. IMR – 52.6 to 13.2/1000 livebirths[45]     2. U5MR- 63.1 to 14.8/1000 livebirths[45]     3. MMR- 184 to 58/100000 livebirths[45]     4. HIV prevalence changed from 0.3% In 1990 to 0.5% In 2017[46] | Indicators (1990s to 2017)   - - 1. IMR- 9.4 to 5.7/1000 live births[47]     2. U5MR- 11.2 to 6.6/1000 live births[48]     3. MMR- 7.6 to 15/100000 live births[49]     4. HIV prevalence remained 0.34% (850,000 out of 250 mil in 1990 to 1.1 mil out of 323.4 mill in 2016)[50, 51] |  |
| 1. Agriculture and food supply: Export and local   Consumption | Agriculture as a main source of export [52], several food security and child nutrition programs as part of SDGs 1&2[53] (e.g. National Nutrition Program). Overall food security index was 39.4% in 2012 and 36% in 2017 (ranked 100^th^ out of 113 countries)[54] | Main export is agriculture & crude petroleum; established School Food Program[55], conditional cash transfer program (Bolsa Família) [43]; overall food security index was 65.8% in 2012 and 68.4% in 2017 (ranked 39^th^ out of 113 countries)[54] | Main export refined petroleum & cars, planes, helicopters & space craft; Significant gap in food supply compared to the Federal Dietary Guidance; Overall food security index was 85.6% in 2012 and 85% in 2017 (ranked 3^rd^ out of 113 countries)[54, 56] |  |
| 1. Employment: Conditions of work | Relative rate of unemployment and child labour reduced, paid maternity leave, pension scheme for older people through employer contribution [52, 57, 58] | Increased job stability and wages, reduced unemployment, reduced child labour and slavery, paid maternity leave, pension scheme for older people [59, 60] | Significant underemployment and inequalities relative to other OECD countries, no change of federal minimum wage since 1996, lowest paid sickness leave and public pensions compared to other high-income countries (e.g. Sweden) [56, 61] |  |
| 1. Income: Wealth & poverty levels | Poverty reduced, purchasing power increased, introduction of social security programs such as health care finance & community-based health insurance, relatively equitable access to resources[39-42], Gini coefficient was 0.35 in 2015 [58] | Improved social welfare, reduced poverty and inequality, government introduced cash transfer program (Bolsa Familia) program and Unified Health System (SUS) [43] [17, 29]. Gini coefficient was 0.074 in 2014 [62] | Weak social security programs, high rate of income inequalities (Gini index rose by 4%, top 1% of population accounts for 40% of nation’s wealth), relative poverty and child poverty (20% of all children estimated to be living in poverty) [63]. Gini coefficient was 0.415 in 2016 [62] |  |
| 1. Housing | Improved housing conditions, access to safe water & ratification of public health regulations [64, 65] | Improved sanitation, access to safe water & housing conditions [31] | Low affordability and inequitable access to housing, significant level of homelessness and housing instability, limited coverage of water supply, and loose public health regulations compared to other developed countries [66, 67] |  |
| i. Housing supply | Improved housing supply through 'Condominium' (a government loan-based housing program), expanded urbanization to address supply shortage, established Urban Development Package (e.g. Integrated Housing Development Program) [64, 65] | Improved housing supply with adequate electricity, sewage disposal, population living in informal settlements decreased from 37% in 1990 to 22% in 2014 [31] | Limited affordable housing compared to other OECD countries, significant cost burden among households; underfunding of the National Housing Trust Fund [66, 67] |  |
| ii. Access to safe water | Access to drinking water was 65% (increased by 800%), increases in quantity & reduced distance to collect water [68] | Equitable access to safe water coverage increased to 98% [31] | Inequitable access to safe water in low income and minority families, limited scope of the Healthy Hunger-Free Kids Act program [69, 70] |  |
| iii. Effective urban planning & healthy infrastructure | Urban Good Governance Package (promoting effective urban planning, improving infrastructure, justice reform and other packages) [65]; WHO Framework Convention on Tobacco Control was ratified in 2014 [71]; excise tax bill on alcohol and tobacco on November 2019 [72] | WHO Framework Convention on Tobacco Control was ratified in 2005 [73] | Non-ratification of WHO Framework Convention on Tobacco Control [74], less regulated markets (e.g. uneven restrictions on advertising of unhealthy products) [75-77] |  |
| i. Environment: sustainable practices & pollution | Ethiopian Environmental Protection Authority established in 1994, Climate resilient green economy strategy including the following proclamations: Environment Impact Assessment Proclamation, Pollution Control Proclamation, Industrial Waste Handling [78, 79] | Strong environmental institutions, creation of Special Secretariat of Environment (SEMA), National Environmental System (SISNAMA), the National Environmental Council (CONAMA) and the Brazilian Institute of Environment and Renewable Natural Resources (IBAMA)[80] | Little action towards a sustainable environment, highest per capita C0_2_ emissions and oil use, some limited environmental protection through Environmental Protection Agency, Clean Air Act, and Clean Water Act[81-83] |  |

FDI: foreign direct investments; GDP: gross domestic product; HIV: human immune-deficiency virus; IMR: infant mortality rate; MMR: maternal mortality ratio; NGOs: non-governmental organisations; OECD: Organisation for Economic Cooperation and Development; PHC: primary health care; PPP: purchasing power parity; SDGs: sustainable development goals; U5MR: under-five mortality rate

**References**

1. **Ethiopia: Life expectancy** [<https://www.theglobaleconomy.com/Ethiopia/Life_expectancy/>]

2. **Brazil Life Expectancy 1950-2019** [<https://www.macrotrends.net/countries/BRA/brazil/life-expectancy> ]

3. **Life expectancy at birth, total (years)** [<https://data.worldbank.org/indicator/SP.DYN.LE00.IN>]

4. Preston SH. The Changing Relation between Mortality and level of Economic Development. Population Studies. 1975; 29(2):231-248.

5. **GDP per capita and GDP (current US$)** [<https://data.worldbank.org/indicator/NY.GDP.PCAP.CD>]

6. **Land area (sq. km)** [<https://data.worldbank.org/indicator/AG.LND.TOTL.K2>]

7. Erwin van V: **Perpetuating power: Ethiopia’s political settlement and the organization of security**. In: *Dynamics of political power in Ethiopia: past and present.* The Hague, The Netherlands: The Clingendael Institute; 2016.

8. de Waal A. The theory and practice of Meles Zenawi. African Affairs. 2012; 112(446):148-155.

9. Emanuele F. Developmental state, economic transformation and social diversification in Ethiopia. ISPI Analysis: Instituto per gli Studi di Politica Internazionale. 2013(163):3.

10. Cason JW, Power TJ. Presidentialization, Pluralization, and the Rollback of Itamaraty: Explaining Change in Brazilian Foreign Policy Making in the Cardoso-Lula Era. International Political Science Review. 2009; 30(2):117-140.

11. Ward K, England K: **Introduction: Reading Neoliberalization**. In: *Neoliberalization: States, Networks, People.* edn. Edited by England K, Ward K. Oxford, UK: Blackwell Publishing; 2007.

12. **School enrollment, primary, female (% gross) - Ethiopia, Brazil, United States** [<https://data.worldbank.org/indicator/SE.PRM.ENRR.FE?locations=ET-BR-US>]

13. Dickman SL, Himmelstein DU, Woolhandler S. Inequality and the health-care system in the USA. Lancet. 2017; 389(10077):1431-1441.

14. Karin H, Dehab B, Asegedech B, Anbesu B, Nuri K. Taking Stock of Girls' Education in Ethiopia: Preparing for ESD P III. 2005.

15. **Ethiopia: Statistics** [<http://www.unicef.org/infobycountry/ethiopia_statistics.html>]

16. **Percentage of women in national parliaments** [<https://data.ipu.org/women-ranking?month=9&year=2019>]

17. Paim J, Travassos C, Almeida C, Bahia L, Macinko J. The Brazilian health system: history, advances, and challenges. Lancet. 2011; 377(9779):1778-1797.

18. Franklin R: **Anuario Estatistico Do Brasil -1982**. In*.*; 1982.

19. Franklin R: **Brasil, Ministério do Planejamento, Orçamento e Gestão. Instituto Brasileiro de Geografia e Estatísitca. Anuário Estatístico do Brasi**. In*.*; 2017.

20. Jou J, Kozhimannil KB, Abraham JM, Blewett LA, McGovern PM. Paid Maternity Leave in the United States: Associations with Maternal and Infant Health. Matern Child Health J. 2018; 22(2):216-225.

21. **Maternity Leave in the United States: Facts You Need to Know** [<https://www.healthline.com/health/pregnancy/united-states-maternity-leave-facts#1>]

22. FDRE: **Health Sector Development Program IV 2010/11 – 2014/15** In*.*, vol. Final Draft. Addis Ababa: Minstory of Health; 2010.

23. Banteyerga H. Ethiopia's health extension program: improving health through community involvement. MEDICC Rev. 2011; 13(3):46-49.

24. Medhanyie A, Spigt M, Kifle Y, Schaay N, Sanders D, Blanco R *et al*. The role of health extension workers in improving utilization of maternal health services in rural areas in Ethiopia: a cross sectional study. BMC health services research. 2012; 12:352.

25. Ruducha J, Mann C, Singh NS, Gemebo TD, Tessema NS, Baschieri A *et al*. How Ethiopia achieved Millennium Development Goal 4 through multisectoral interventions: a Countdown to 2015 case study. The Lancet Global health. 2017; 5(11):e1142-e1151.

26. Doherty T, Rohde S, Besada D, Kerber K, Manda S, Loveday M *et al*. Reduction in child mortality in Ethiopia: analysis of data from demographic and health surveys. Journal of global health. 2016; 6(2):020401-020401.

27. CSA, ICF: **Ethiopian Demographic Health Survey 2016**. In*.* Addis Ababa and Calverton: Central Statistical Agency (Ethiopia) and ICF International; 2018: 36-41.

28. Girum T, Wasie A, Worku A. Trend of HIV/AIDS for the last 26 years and predicting achievement of the 90–90-90 HIV prevention targets by 2020 in Ethiopia: a time series analysis. BMC infectious diseases. 2018; 18(1):320.

29. Castro MC, Massuda A, Almeida G, Menezes-Filho NA, Andrade MV, de Souza Noronha KVM *et al*. Brazil's unified health system: the first 30 years and prospects for the future. Lancet. 2019; 394(10195):345-356.

30. Giugliani C, Harzheim E, Duncan MS, Duncan BB. Effectiveness of community health workers in Brazil: a systematic review. The Journal of ambulatory care management. 2011; 34(4):326-338.

31. Macinko J, Harris MJ. Brazil's family health strategy--delivering community-based primary care in a universal health system. The New England journal of medicine. 2015; 372(23):2177-2181.

32. Neves RG, Flores TR, Duro SMS, Nunes BP, Tomasi E. Time trend of Family Health Strategy coverage in Brazil, its Regions and Federative Units, 2006-2016. Epidemiologia e servicos de saude : revista do Sistema Unico de Saude do Brasil. 2018; 27(3):e2017170.

33. Woolf S, Aron L: **Shorter lives, poorer health: US health in international perspective**. In*.* Washington DC: National Academies; 2013.

34. Wilkinson R, Pickett K. The Spirit Level: Why More Equal Societies Almost Always Do Better. United Kingdom: Bloomsbury Press; 2010.

35. Assefa Y, Tesfaye D, Damme WV, Hill PS. Effectiveness and sustainability of a diagonal investment approach to strengthen the primary health-care system in Ethiopia. Lancet. 2018; 392(10156):1473-1481.

36. Ibrhim MA, Demissie M, Medhanyie AA, Worku A, Berhane Y. Reasons for low level of skilled birth attendance in Afar pastoralist community, North East Ethiopia: a qualitative exploration. Pan Afr Med J. 2018; 30:51.

37. **Health Care Policy Is Undermined by Voting Barriers** [<https://tcf.org/content/report/health-care-policy-undermined-voting-barriers/?session=1&session=1>]

38. Ziegenfuss JK, Davern M, Blewett LA. Access to health care and voting behavior in the United States. J Health Care Poor Underserved. 2008; 19(3):731-742.

39. Ali EE. Health Care Financing in Ethiopia: Implications on Access to Essential Medicines. Value Health Reg Issues. 2014; 4:37-40.

40. Hailu Z: **Health Care Financing Reform in Ethiopia: Improving Quality and Equity**. In*.* Washington: USAID; 2012.

41. Mebratie AD, Sparrow R, Yilma Z, Alemu G, Bedi AS. Enrollment in Ethiopia’s Community-Based Health Insurance Scheme. World Development. 2015; 74:58-76.

42. Mebratie AD, Sparrow R, Yilma Z, Abebaw D, Alemu G, Bedi AS. The impact of Ethiopia's pilot community based health insurance scheme on healthcare utilization and cost of care. Social science & medicine (1982). 2019; 220:112-119.

43. Rasella D, Aquino R, Santos CA, Paes-Sousa R, Barreto ML. Effect of a conditional cash transfer programme on childhood mortality: a nationwide analysis of Brazilian municipalities. The Lancet. 2013; 382(9886):57-64.

44. Smith JC, Medalia C. Health insurance coverage in the United States: 2013: US Department of Commerce, Economics and Statistics Administration, Bureau …; 2014.

45. Leal MDC, Szwarcwald CL, Almeida PVB, Aquino EML, Barreto ML, Barros F *et al*. Reproductive, maternal, neonatal and child health in the 30 years since the creation of the Unified Health System (SUS). Ciencia & saude coletiva. 2018; 23(6):1915-1928.

46. **Prevalence of HIV, total (% of population ages 15-49)** [<https://data.worldbank.org/indicator/SH.DYN.AIDS.ZS>]

47. Riddell CA, Harper S, Kaufman JS. Trends in Differences in US Mortality Rates Between Black and White Infants. JAMA pediatrics. 2017; 171(9):911-913.

48. **Mortality rate, under-5 (per 1,000 live births)** [<https://data.worldbank.org/indicator/SH.DYN.MORT>]

49. **Maternal mortality ratio (modeled estimate, per 100,000 live births) - United States** [<https://data.worldbank.org/indicator/SH.STA.MMRT?locations=US>]

50. CDC: **Estimated HIV incidence and prevalence in the United States, 2010–2016.** . In: *HIV Surveillance Supplemental Report 2019.* vol. 24: Centers for Disease Control and Prevention; 2019.

51. CDC. HIV surveillance--United States, 1981-2008. MMWR Morbidity and mortality weekly report. 2011; 60(21):689-693.

52. **Ethiopia: Unemployment, total (% of total labor force) (modeled ILO estimate)** [<https://data.worldbank.org/indicator/SL.UEM.TOTL.ZS?end=2018&locations=ET&start=1991&view=chart>]

53. **National Network of Positive Women Ethiopians (NNPWE)** [<https://www.nnpwe.org/>]

54. **Global Food Security Index: Rankings and trends** [<https://foodsecurityindex.eiu.com/Index>]

55. Teo C. The partnership between the Brazilian School Feeding Program and family farming: a way for reducing ultra-processed foods in school meals. Public Health Nutr. 2018; 21(1):230-237.

56. Woolf S, Aron L. US health in international perspective: Shorter lives, poorer health. Washington DC: National Academies Press; 2013.

57. Chalachew Getahun D. The urban informal economy in Ethiopia: theory and empirical evidence. Eastern Africa Social Science Research Review. 2018; 34(1).

58. **GINI index (World Bank Estimate)** [<https://www.ceicdata.com/en/ethiopia/poverty/et-gini-coefficient-gini-index-world-bank-estimate>]

59. Francisco A, Joan B, Antía C, Yucel D, Chamberlain D, Magdalena E *et al*: **Employment Conditions and Health Inequalities: Final Report to the WHO Commission on Social Determinants of Health (CSDH): Employment Conditions Knowledge Network (EMCONET)**. In*.* Ontario, Canada; 2007.

60. Furio R, Marco M, Irina K, Nihan K, Scott L: **Understanding the Brazilian success in reducing child labour: empirical evidence and policy lessons**. In*.*; 2011.

61. Beckfield J, Bambra C. Shorter lives in stingier states: Social policy shortcomings help explain the US mortality disadvantage. Social science & medicine. 2016; 171:30-38.

62. **GINI index (World Bank estimate)** [<https://data.worldbank.org/indicator/SI.POV.GINI>]

63. Edward W: **Household wealth trends in the United States, 1962 to 2016: has middle class wealth recovered?** In*.* Massachusetts: NATIONAL BUREAU OF ECONOMIC RESEARCH; 2017.

64. Tameru W: **Affordable Houses for Middle and Low Income Group in Ethiopia: Self help housing with innovative construction technology** In*.* Addis Ababa: Housing Development Department, Ministry of Works and Urban Development, Ethiopia 2009.

65. Kassahun S, Tiwari A. Urban Development in Ethiopia: Challenges and Policy Responses. The IUP Journal of Governance and Public Policy. 2012; 7(1):59-65.

66. HarvardUniversity: **The State of The Nation’s (United States) Housing 2016—Housing challenges**. In*.* Massachusetts Harvard University; 2016.

67. Kathie S. A Brief Analysis of the National Housing Trust Fund. Journal of Affordable Housing & Community Development Law. 2008; 18(1):13-26.

68. **Ethiopia meets MDG target on safe water supply** [<https://www.afro.who.int/news/ethiopia-meets-mdg-target-safe-water-supply>]

69. Patel AI, Schmidt LA. Water Access in the United States: Health Disparities Abound and Solutions Are Urgently Needed. American journal of public health. 2017; 107(9):1354-1356.

70. Erin R, Jeff H, Daniel I, Kyle L: **An Overview of Clean Water Access Challenges in the United States**. In*.*: Environmental Finance Center at the University of North Carolina, Chapel Hill; 2017.

71. **Ethiopia Ratifies WHO Framework Convention on Tobacco Control** [<https://afro.who.int/news/ethiopia-ratifies-who-framework-convention-tobacco-control>]

72. **Council Of Ministers Approves Investment, Privatization Bills** [[**https://ethiopianmonitor.com/2019/12/02/council-of-ministers-approves-investment-privatization-bills/**](https://ethiopianmonitor.com/2019/12/02/council-of-ministers-approves-investment-privatization-bills/)]

73. **Brazil—WHO Framework Convention on Tobacco Control** [<https://www.inca.gov.br/en/observatory-of-the-national-policy-on-tobacco-control/who-framework-convention-tobacco-control>]

74. **Framework Convention on Tobacco Control** [<http://www.who.int/tobacco/framework/WHO_FCTC_english.pdf>]

75. Thomson K, Hillier-Brown F, Todd A, McNamara C, Huijts T, Bambra C. The effects of public health policies on health inequalities in high-income countries: an umbrella review. BMC public health. 2018; 18(1):869.

76. De Vogli R, Kouvonen A, Gimeno D. The influence of market deregulation on fast food consumption and body mass index: a cross-national time series analysis. Bulletin of the World Health Organization. 2014; 92:99-107A.

77. Freudenberg N. Lethal but legal: corporations, consumption, and protecting public health: Oxford University Press; 2014.

78. FDRE: **Ethiopia’s Climate-Resilient Green Economy: Green economy strategy** In*.*; 2011.

79. EPA: **Federal Democratic Republic of Ethiopia: Guideline Document for Environmental Impact Assessment, Environmental Protection Authority, Addis Ababa, Ethiopia**. In*.*; 2000.

80. Guilherme N, Vanessa L: **Brazilian Environmental Policies and Issues**. In*.*; 2016.

81. Michael H, Liana W, Ruth P, Aysin D-H, Silvia S-N, Julie D *et al*. Environmental Sustainability: A Case of Policy Implementation Failure? Sustainability. 2017; 9(2):165.

82. **People using at least basic drinking water services (% of population) - United States** [<https://data.worldbank.org/indicator/SH.H2O.BASW.ZS?locations=US>]

83. VanDerslice J. Drinking water infrastructure and environmental disparities: evidence and methodological considerations. American journal of public health. 2011; 101 Suppl 1(Suppl 1):S109-S114.

1. Calculated from a Preston curve with 2018 GDP per capita, International $ PPP and 2018 life expectancy at birth data, where predicted life expectancy = 5.39 x ln(GDP) + 21.8. GDP = Gross Domestic Product, PPP = Purchasing Power Parity. [↑](#footnote-ref-1)
2. Life expectancy (LE) to GDP weight status is calculated by subtracting expected LE from LE at birth, PAW= Punching Above Weight; PBW= Punching Below Weight [↑](#footnote-ref-2)
3. Dependency ratio, an age-population ratio, is the proportion of people who are not in the labour force aged between 0 to 14 and 65+) to people who are in the productive or labour force aged between 15 to 64. [↑](#footnote-ref-3)
4. Gross enrolment ratio (GER) is the number of students enrolled in a given level of education regardless of age divided by the population of the age group which officially corresponds to the given level of education, and multiply the result by 100. A high GER generally indicates a high degree of participation, whether the pupils belong to the official age group or not. A GER value approaching or exceeding 100% indicates that a country is, in principle, able to accommodate all of its school-age population, but it does not indicate the proportion already enrolled. [↑](#footnote-ref-4)
